# Preoperative Simulation of Intraoperative Findings in Surgical Clipping of Posterior Communicating Artery Aneurysms Using T2-Weighted 3D Images

**DOI:** 10.7759/cureus.66851

**Published:** 2024-08-14

**Authors:** Yushi Nagano, Taichi Ikedo, Koji Shimonaga, Yuji Kushi, Eika Hamano, Hirotoshi Imamura, Hisae Mori, Ryosuke Hanaya, Koji Iihara, Hiroharu Kataoka

**Affiliations:** 1 Department of Neurosurgery, National Cerebral and Cardiovascular Center, Suita, JPN; 2 Department of Neurosurgery, Graduate School of Medical and Dental Sciences, Kagoshima University, Kagoshima, JPN; 3 Department of Neurosurgery, Kyoto University, Kyoto, JPN

**Keywords:** t2-weighted 3d images with an se-type sequence, surgical clipping, preoperative simulation, posterior communicating artery aneurysm, intracranial aneurysm

## Abstract

Background: Tentorium resection and detachment from the oculomotor nerve are sometimes required for surgical clipping of unruptured posterior communicating artery (PCoA) aneurysms. Using T2-weighted 3D images, we aimed to identify the preoperative radiological features required to determine the necessity of these additional procedures.

Methods: We reviewed 30 patients with unruptured PCoA aneurysms who underwent surgical clipping and preoperative simulation using T2-weighted 3D images for measurement of the distance between the tentorium and aneurysm. Aneurysms were classified into superior type (superior to the tentorium) and inferior type (inferior to the tentorium).

Results: Seven patients (23%) underwent tentorium resection; all had the inferior type (superior vs. inferior, 0% vs. 33%, p = 0.071). In the 21 patients with the inferior type, the distance from the tentorium to the aneurysmal neck was 2.2 ± 1.1 mm and 0.0 ± 0.5 mm without and with tentorium resection (p < 0.01), respectively. An optimal cutoff value of ≤ +0.84 mm was identified for tentorium resection (area under the curve (AUC) = 0.96). Furthermore, 17 patients (57%) showed tight aneurysm attachment to the oculomotor nerve; all had the inferior type (0% vs. 81%, p < 0.01). The distance from the aneurysm tip to the tentorium was 1.1 ± 1.2 mm and -1.7 ± 1.4 mm without and with attachment (p < 0.01). The optimal cutoff value was ≤ +0.45 mm (AUC = 0.92).

Conclusions: Measurement of the distance between the tentorium and aneurysmal neck or tip with T2-weighted 3D images is effective for preoperative simulation for surgical clipping of PCoA aneurysms.

## Introduction

Microsurgical clipping is an established intervention for unruptured intracranial aneurysms. Total detachment of the aneurysmal neck and dome from surrounding tissues is sometimes performed to facilitate complete occlusion and ensure a low complication rate. In cases of microsurgical clipping for posterior communicating artery (PCoA) aneurysms, several key anatomical structures must be considered, including the oculomotor nerve, anterior choroidal artery, temporal lobe, anterior clinoid process (ACP), and anterior petroclinoid fold (APF), which is part of the tentorium [1]. Preoperative enhanced computed tomography (CT) could predict the necessity for anterior clinoidectomy to secure sufficient space for microscopic manipulation in the microsurgical clipping of PCoA aneurysms [2-5]. Niibo et al. further showed that a short distance between the proximal aneurysmal neck and the tip of the ACP and the presence of calcification at the ophthalmic segment of the internal carotid artery (ICA) on preoperative CT angiography was associated with difficulty of achieving proximal vascular control [5]. Other important procedures required in the microsurgical clipping of PCoA aneurysms include resection of the tentorium to visualize the aneurysmal neck and detachment of the aneurysm from the oculomotor nerve [3,6,7]. However, accurately determining the necessity of these procedures is challenging using only enhanced CT images, as this technique cannot visualize the tentorium and oculomotor nerves.

T2-weighted 3D images with a spin echo (SE)-type sequence, a 3D fast SE method that collects data while changing the refocus flip angle, is a relatively new imaging method for magnetic resonance (MR) hydrography and has reportedly provided cisternal images with preserved contrast and reduced artifact [8]. This technique has been beneficial in the spinal cord area, particularly in identifying intracranial denticulate ligaments and cranial nerves [9,10]. T2-weighted 3D imaging with an SE-type sequence can visualize the tentorium and oculomotor nerves; however, few studies evaluated the benefits of T2-weighted 3D images in the preoperative simulation of tentorial resection and detachment of the oculomotor nerve in microsurgical clipping of PCoA aneurysms.

Therefore, the present study aimed to use preoperative T2-weighted 3D images in identifying the preoperative radiological features required to determine the necessity of intraoperative tentorium resection and to visualize the adhesion between the oculomotor nerve and the aneurysm.

## Materials and methods

Patient population

Between January 2012 and January 2022, 92 patients with unruptured PCoA aneurysms underwent surgical clipping in our institute. Among these, we included 32 patients who underwent T2-weighted 3D imaging with an SE-type sequence such as T2 sampling perfection with optimized contrast application using different flip angle evolutions (SPACE; Siemens, Erlangen, Germany), T2 volume isotropic turbo spin-echo acquisition (VISTA; Philips, Amsterdam, Netherlands), Cube (General Electric, Connecticut, USA), and iso 3D Fast Spin Echo (FSE, Canon, Tokyo, Japan). The parameters for coronal sections were as follows: repetition time 2000-2300 ms, echo time 100-173 ms, slice thickness 0.3-0.6 mm, matrix (0.26-0.52 mm) × (0.26-0.52 mm) × 0.6 mm, flip angle 90°, scan duration 3.9-5.2mm, and field of view 200 mm. Two patients who had undergone anterior clinoidectomy for proximal vascular control were excluded. This was because this study was designed to simulate the tentorium resection to secure working space for clipping, and therefore, the cases in which the tentorium was partially resected in conjunction with the anterior clinoidectomy were not included. We retrospectively reviewed baseline characteristics, radiological findings, and operative findings for the included 30 patients. This study was approved by the institutional review board of our institute. The requirement for informed consent was waived because this was a retrospective study.

Procedures of the surgical clipping for PCoA aneurysms

All patients underwent surgery with ipsilateral transsylvian approaches under general anesthesia. Frontotemporal craniotomy was performed, followed by extradural removal of the sphenoid ridge. The frontal and temporal lobes were separated with a sharp dissection of the arachnoid membrane, and a wide opening of the Sylvian fissure was achieved to visualize the ICA and the proximal portion of the middle cerebral artery. Proximal control of the ICA was obtained by opening the opticocarotid cistern. If the space around the aneurysmal neck was insufficient for safe clipping due to the presence of tentorium, resection of the tentorium was performed. In cases of tight attachment of the aneurysm to the ipsilateral oculomotor nerve, sharp dissection of the arachnoid between the aneurysm and oculomotor nerve was performed to ensure sufficient mobility of the aneurysm for clipping. Tight attachment was defined as the adhesion between the aneurysm and oculomotor nerve detached using surgical tools. After total detachment of the PCoA aneurysm from the surrounding structures, the clip was applied to the aneurysmal neck. Complete clipping and preservation of the arteries were confirmed by indocyanine green video angiography.

Data collection

The patients’ medical records were retrospectively reviewed to obtain relevant clinical information, including age, sex, symptoms, and surgery date. Surgical findings with or without resection of the tentorium and the adhesion between the aneurysms and the oculomotor nerves were judged using operative records.

Measurement of the aneurysmal location using T2-weighted 3D imaging

**Figure 1:**
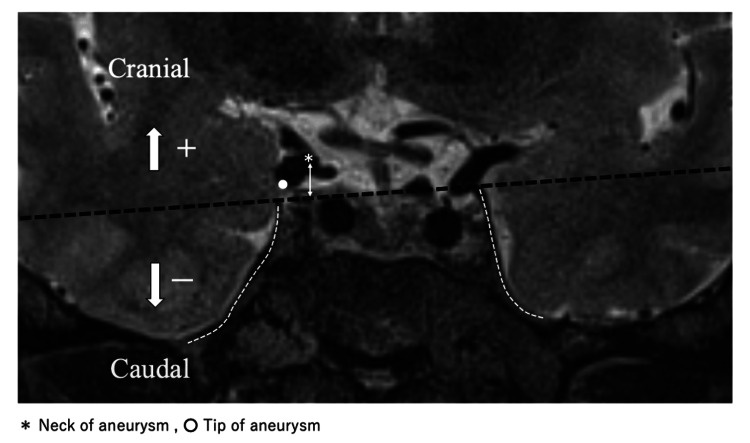
Measurement of the distance from the tentorium to the aneurysmal neck or tip A coronal image of the preoperative T2-weighted 3D image is shown. The bilateral tentorium is indicated with a white dot-dash line. A line is drawn connecting both bilateral tips of the tentorium (black dot-dash line). Aneurysmal projections are classified into two types: superior to tentorium (superior type) and inferior to tentorium (inferior type). If they are located X mm cranial to the line, the distance is identified as + X mm. If they are located Y mm caudal to the line, the distance is identified as - Y mm. An asterisk indicates the aneurysmal neck. A white dot indicates the tip of the aneurysm. A white double arrow shows the distance between the tentorium and the aneurysmal neck.

Coronal sections of the preoperative T2-weighted 3D images were used for visualizing the tentorium, oculomotor nerve, and aneurysms (Figure 1).

In addition, we drew a line (the tentorium line) connecting both bilateral tops of the tentorium. Aneurysmal location was classified into two types: superior to the tentorium (superior type) and inferior to the tentorium (inferior type). When the dome of the aneurysm was located superior or inferior to the tentorium line (Figure 1), the aneurysm was defined as the superior or inferior type, respectively. If the dome of the aneurysm was located both superior and inferior to the tentorium, we identified the aneurysm as inferior. Subsequently, we measured the distance between the tentorium line and the aneurysmal neck of the PCoA or the tip of the aneurysm. The tip of the aneurysm was defined as the lowest point in the coronal section in the present study. Thus, the coronal section including the lowest point of the aneurysm was chosen for the distance measurement. The tentorial line was drawn in the same coronal slice. If the tips were located X mm cranial to the tentorium line, the distance was identified as + X mm. If they were located Y mm caudal to the tentorium line, the distance was judged as - Y mm (Figure 1). Similarly, the distance between the aneurysmal neck and tentorium line was measured on the coronal section including the aneurysmal neck.

Statistical analysis

Statistical analyses were conducted using commercially available statistics software (Prism version 9.1.2; GraphPad, Boston, MA, USA). Univariate analyses were performed using the Mann-Whitney U-test for quantitative variables to assess the differences in the estimated data, whereas Fisher’s exact test was used for the categorical variables. The results were considered significant at p < 0.05. Furthermore, receiver operating characteristic (ROC) analysis was used to identify the best cutoff distance for determining whether distance affected the necessity for intraoperative resection of the tentorium and aneurysm attachment to the oculomotor.

## Results

Patient characteristics

The clinical and radiological characteristics of the 30 patients who underwent clipping surgery for PCoA aneurysms are summarized in Table 1.

**Table 1:** Baseline characteristics of the 30 patients who underwent surgical clipping for PCoA aneurysms Data are presented as the mean ± standard deviation or number of patients (%). PCoA: posterior communicating artery

Parameters	Patients (n=30)
Age, years	67 ± 11
Sex (female, %)	24 (80)
Aneurysm location (right, %)	12 (40)
Classification of aneurysms (superior, %)	9 (30)
Bleb (%)	19 (63)
Aneurysm maximum diameter, mm	6.5 ± 2.7
Aneurysm dome, mm	5.4 ± 2.3
Aneurysm height, mm	4.7 ± 2.6
Aneurysm neck, mm	3.8 ± 1.3
Tentorium - neck of aneurysm	2.0 ± 1.9
Tentorium - tip of aneurysm	-0.51 ± 2.3

Patient ages ranged from 40 to 93 years (mean ± SD, 67 ± 11 years), 24 patients (80%) were female, and 12 aneurysms (40%) were on the right side. Overall, 21 aneurysms (70%) were classified as the inferior type, and nine aneurysms (30%) were the superior type. Additionally, 19 aneurysms (63%) had blebs, and the maximum diameter of PCoA aneurysms ranged from 1.4 to 15 mm (6.5 ± 2.7 mm). On the coronal preoperative T2-weighted 3D images, the distance between the tentorium and aneurysmal neck ranged from -0.89 mm to 8.0 mm (2.0 ± 1.9 mm), and the distance between the tentorium and aneurysmal tip ranged from -4.1 mm to 7.4 mm (-0.5 ± 2.3 mm).

Tentorium resection

Of the 30 patients, seven (23%) underwent tentorium resection for proximal vascular control or for visualization of the space between surrounding tissues and the aneurysmal neck to achieve complete clipping. All these cases were classified as the inferior type (superior vs. inferior, 0% vs. 33%, p = 0.071). In the 21 patients with inferior type, the distance from the tentorium to the aneurysmal neck was 2.2 ± 1.1 mm in cases without tentorium resection and 0.0 ± 0.5 mm in cases with tentorium resection (p < 0.01, Table 2).

**Table 2:** Univariate analysis of factors influencing tentorium resection in unruptured posterior communicating artery aneurysm surgery in 21 patients with the inferior type Data are presented as the mean ± standard deviation or number of patients (%). *Variables significantly related to tentorium resection.

Parameters	(+) n = 7	(-) n = 14	p-value
Age, years	67 ± 14	69 ± 4.6	0.66
Sex (female, %)	5 (71)	13 (93)	0.42
Aneurysm location (right, %)	4 (57)	4 (29)	0.35
Bleb (%)	6 (86)	10 (67)	0.62
Aneurysm maximum diameter, mm	7.0 ± 1.3	7.0 ± 2.9	0.99
Aneurysm dome, mm	5.7 ± 1.7	5.7 ± 2.3	0.98
Aneurysm height, mm	4.7 ± 1.2	5.5 ± 2.9	0.52
Aneurysm neck, mm	4.4 ± 1.2	3.9 ± 0.93	0.37
Tentorium - neck of aneurysm	0.0 ± 0.5	2.2 ± 1.1	<0.01*
Tentorium - tip of aneurysm	-1.5 ± 1.3	-1.0 ± 1.9	0.55

**Figure 2:**
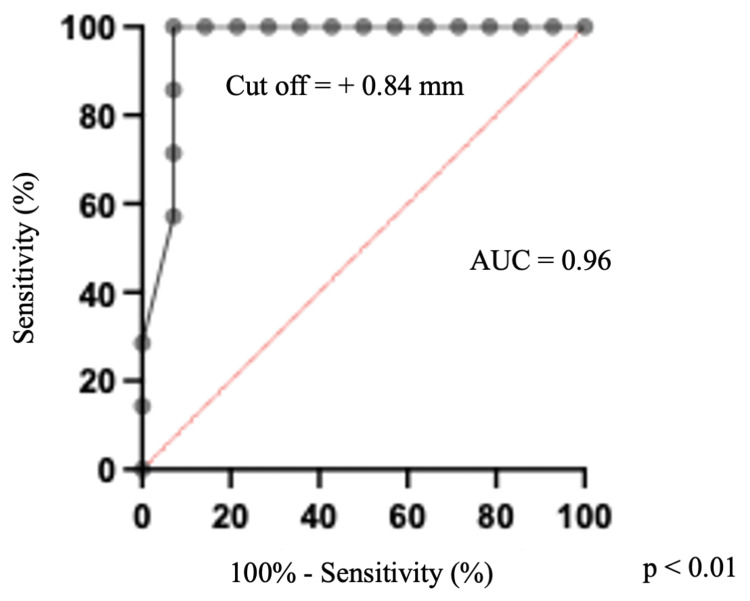
Receiver operating characteristic curve for the prediction of tentorium resection Receiver operating characteristic curve analysis showing the optimal cut-off value to predict the need for tentorium resection. The cutoff value of the distance from the tentorium to the PCoA aneurysmal neck yielding optimal sensitivity and specificity (100% and 92.9%, respectively) is + 0.84 mm. AUC: area under the curve; PCoA, posterior communicating artery

Other parameters including aneurysmal size or distance from the tentorium to the aneurysmal tip were not associated with tentorium resection (Table 2). ROC curve analysis revealed that the distance from the tentorium to the aneurysmal neck of ≤ +0.84 mm was the optimal cutoff value for establishing the necessity of tentorium resection (area under the curve (AUC): 0.96, cutoff value sensitivity: 100%, specificity: 93%; Figure 2).

Adhesion between the oculomotor nerve and aneurysm

Of the 30 cases, 17 (57%) had tight aneurysm attachment to the oculomotor nerve, all of which occurred in aneurysms classified as the inferior type (superior vs. inferior, 0% vs. 81%, p < 0.01). In the 21 patients with the inferior type, the distance from the tip of the aneurysm to the tentorium was 1.1 ± 1.2 mm in cases without tight attachment to the oculomotor nerve, and -1.7 ± 1.4 mm in cases with tight attachment (p < 0.01, Table 3).

**Table 3:** Univariate analysis of factors influencing attachment of the oculomotor nerve to aneurysms in unruptured posterior communicating artery aneurysm surgery in 21 patients with the inferior type Data are presented as the mean ± standard deviation or number of patients (%). *Variables significantly related to attachment of the oculomotor nerve to aneurysms.

Parameters	(+) n = 17	(-) n = 4	p-value
Age, years	69 ± 10	68 ± 3.8	0.96
Sex (female, %)	15 (88)	3 (75)	0.49
Aneurysm location (right, %)	7 (41)	1 (25)	0.99
Bleb (%)	13 (76)	3 (75)	0.99
Aneurysm maximum diameter, mm	7.2 ± 2.7	6.1 ± 1.1	0.48
Aneurysm dome, mm	5.7 ± 2.3	5.6 ± 0.4	0.90
Aneurysm height, mm	5.5 ± 2.7	4.0 ± 0.71	0.29
Aneurysm neck, mm	4.0 ± 1.1	4.5 ± 0.32	0.36
Tentorium - neck of aneurysm	1.2 ± 1.4	2.8 ± 0.87	0.04*
Tentorium - tip of aneurysm	-1.7 ± 1.4	1.1 ± 1.2	<0.01*

**Figure 3:**
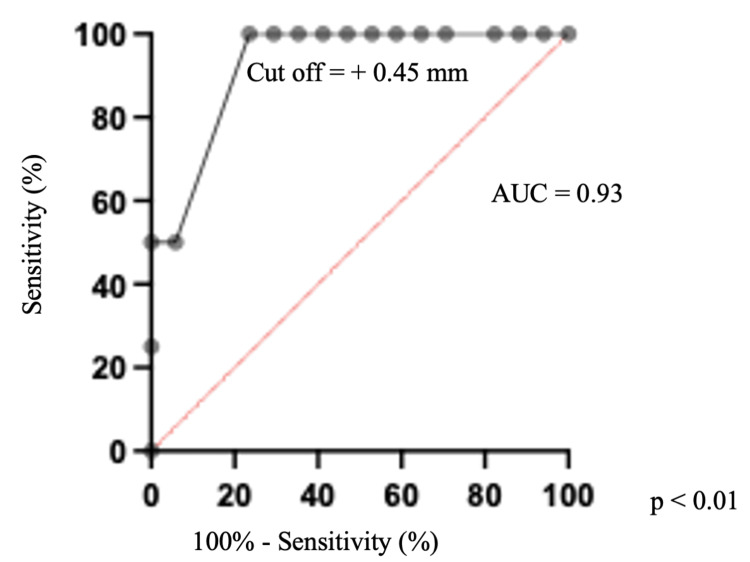
Receiver operating characteristic curve predicting the tight attachment of PCoA aneurysms to the oculomotor nerve Receiver operating characteristic curve analysis showing the optimal cut-off value for predicting the attachment of the aneurysm to the oculomotor nerve. The cutoff value of the distance from the tentorium to the PCoA aneurysmal tip yielding optimal sensitivity and specificity (50% and 94.1%, respectively) is + 0.45 mm. AUC: area under the curve; PCoA, posterior communicating artery

The distance from the tentorium to the aneurysmal neck was also slightly different between the cases with and without adhesion (with adhesion vs. without adhesion, 1.2 ± 1.4 mm vs. 2.8 ± 0.87 mm, p = 0.04). ROC curve analysis revealed that a distance of ≤ +0.45 mm from the tip of the aneurysm to the tentorium was the optimal cutoff value for predicting the tight attachment of the aneurysm to the oculomotor nerve (AUC: 0.92, cutoff value sensitivity: 50%, specificity 94%; Figure 3).

## Discussion

In the present study, we demonstrated that T2-weighted 3D imaging with an SE-type sequence can be used to measure the distance from the tentorium to the aneurysmal neck or tip, which is associated with the intraoperative resection of the tentorium and locate adhesions between the oculomotor nerve and the aneurysm. We further demonstrated the use of T2-weighted 3D imaging with an SE-type sequence for the preoperative simulation of the microsurgical clipping of PCoA aneurysms.

As part of the tentorium, the APF is a ligamentous structure 16.6 ± 2.4 mm in length, extending from the petrous apex to the ACP, and covers the superolateral surface of the ACP [11,12]. Because the APF obstructs the operating field around the aneurysmal neck in some PCoA aneurysm microsurgeries, simple resection of the tentorium is performed in such cases [3,6,7]. Although preoperative prediction of anterior clinoidectomy has been reported [2,4,5], few studies reported using imaging simulation for tentorium resection.

Because CT images cannot be used to visualize the tentorium, we utilized an MRI method, T2-weighted 3D imaging with an SE-type sequence, to visualize the tentorium and enable accurate measurement of the association between the tentorium and PCoA aneurysms. In a similar study using T2-weighted 3D images with an SE-type sequence MRI, a technical note implied the efficacy of an MRI method, fast imaging employing steady-state acquisition. However, a detailed prediction method for the resection of the tentorium was not described. Yoon et al. predicted the intraoperative contact of PCoA aneurysms to the APF using preoperative high-resolution 3D proton density-weighted turbo SE magnetic resonance (PDMR) with a high diagnostic accuracy of 0.90 [13]. However, some cases require tentorial resection to obtain a sufficient operative field around the aneurysmal neck for safe clipping, despite the absence of contact between the PCoA aneurysm and tentorium. Thus, the detection of PCoA aneurysm contact with the APF only is insufficient for predicting intraoperative tentorium resection. We drew a line between the bilateral tentorial edge and measured the distance between the PCoA aneurysm neck and the line. The height of the tentorium is not constant depending on the location. However, the actual need for the tentorium resection is determined by the relationship between the aneurysmal neck and the location of the closest tentorial edge. If the tentorial edge is not far enough from the aneurysmal neck, there is insufficient working space for clipping. For this reason, in the present study, the distance was defined as the space from a line between the bilateral tentorial edge in the same coronal section as the aneurysmal neck or tip, and we succeeded in statistically demonstrating that the measured distance was related to tentorium resection. The distance of ≤ +0.84 mm was found to be the optimal cutoff value for determining the necessity of tentorium resection (AUC: 0.96, cutoff value sensitivity: 100%, specificity: 93%).

Several studies have reported predictors of oculomotor palsy after surgery for PCoA aneurysms [14]; however, few reports have investigated the tight attachment of aneurysms to the oculomotor nerves [13]. In a previous report, postoperative oculomotor nerve palsy had no associations with PCoA aneurysmal size [14]. Consistent with previous studies on oculomotor palsy, we found no correlation between the presence of adhesions and aneurysmal size. This may be because the distance between the ICA and the oculomotor nerve varies widely between individuals, ranging from 3.0 mm to 10.4 mm [14,15]. To investigate the attachment of oculomotor nerves to PCoA aneurysms, we tried to detect oculomotor nerves using T2-weighted 3D images with an SE-type sequence; however, the rate of visualization achieved was only 77% (23/30). Thus, we indirectly measured the distance between the tentorium edge and the PCoA aneurysmal tip to predict the tight attachment of oculomotor nerves and PCoA aneurysms. The optimal cutoff value was set at a distance of > +0.45 mm (AUC: 0.93, cutoff value sensitivity: 50%, specificity: 94%). A previous report predicted the tight attachment of PCoA aneurysms to the oculomotor nerves with PDMR and reported a sensitivity of 92% and specificity of 88%, which is consistent with our results [13]. These results indicate that T2-weighted 3D images are potentially effective for the prediction of the tight attachment of PCoA aneurysms to oculomotor nerves.

The present study has several limitations. First, this study included a small number of patients from a single center. Second, the study protocol had a retrospective design. Third, the 30 microsurgeries in the present study were not performed by a single surgeon. Thus, the indication for tentorium resection is not completely uniform. Fourth, the height of the tentorium is not constant depending on the location. As the setting of the coronal MRI image will be different in each facility, there may be an error in measuring the distance between the tentorial edge and the aneurysm in the application of this method at other institutions. Finally, since other imaging methods including thin-sliced, constructive interference steady-state sequence may delineate the cisternal segment of the oculomotor nerve more frequently than T2-weighted 3D images with SE-type sequence imaging, additional study is required for establishing more accurate diagnostic methods. Although we demonstrated the potential benefits of T2-weighted 3D imaging with an SE-type sequence in the microsurgical clipping of PCoA aneurysms in the present study, large prospective studies are required to validate the impact of this method on preoperative predictions.

## Conclusions

The preoperative relationship between the tentorium and aneurysmal neck and tip was associated with determining the necessity for tentorium resection and the presence of oculomotor nerve and aneurysm attachment. The distance from the tentorium to the aneurysmal neck of ≤ +0.84 mm was the optimal cutoff value for establishing the necessity of tentorium resection. The distance of ≤ +0.45 mm from the tip of the aneurysm to the tentorium was the optimal cutoff value for predicting the tight attachment of the aneurysm to the oculomotor nerve. T2-weighted 3D imaging with an SE-type sequence is a potentially beneficial simulation tool in the microsurgical clipping of PCoA aneurysms.
